# Colorectal gel immersion endoscopic submucosal dissection using the tunneling method

**DOI:** 10.1055/a-2106-2270

**Published:** 2023-07-11

**Authors:** Yuya Nakano, Takeshi Hatanaka, Yoichi Hazama, Yoshiki Tanaka, Yoko Hachisu, Tomoaki Tashima, Toshio Uraoka

**Affiliations:** 1 Department of Gastroenterology, Gunma Saiseikai Maebashi Hospital, Maebashi, Japan; 2 Department of Gastroenterology, Saitama Medical University International Medical Center, Saitama, Japan; 3 Department of Gastroenterology and Hepatology, Graduate School of Medicine, Gunma University, Maebashi, Japan

Colorectal endoscopic submucosal dissection (ESD) is now widely used for the treatment of colorectal tumors using several strategies. The effectiveness of the pocket-creation method combined with saline immersion has been reported with an improved endoscopic visual field and submucosal approach due to the buoyancy of the lesion and reduced the amount of submucosal injection. Its disadvantages include a poor visual field during bleeding and dissipation of coagulation ability with monopolar devices 1 2 . However, gel immersion ESD has been reported to overcome these issues 3 4 5 . As the colorectum has a large lumen and may cause difficulties for gel immersion, it is assumed that a closed lumen such as a pocket or tunnel would facilitate gel immersion; therefore, the strategy of performing gel immersion ESD in combination with the tunneling method was devised.

Herein, we report a combination of gel immersion ESD and tunneling in a colorectal tumor ( Video 1 ). A 71-year-old woman presented with a 50-mm laterally spreading, granular-nodular, mixed-type tumor located in the ascending colon ( Fig. 1 ). Mucosal incision and submucosal dissection during gel immersion ESD were performed using a new electrosurgical knife (GoldKnife T-type 1.5 mm; Micro-Tech Co., Ltd, Nanjing, China), which allowed local injection of hyaluronic acid solution. A suitable approach to the submucosal layer and smooth creation of the tunnel were achieved owing to the buoyancy of the gel ( Fig. 2 a ). Bleeding in the tunnel was clearly visible in the gel, and hemostasis could be quickly and easily secured. After tunnel penetration, mucosal incision and dissection on both sides of the tunnel were also performed smoothly ( Fig. 2 b, c ). The tumor was completely excised without perforation ( Fig. 2 d ). Colorectal gel immersion ESD using the tunneling method may be an alternative approach.

**Video 1** Successful gel immersion colorectal endoscopic submucosal dissection using the tunneling method.


**Fig. 1:**
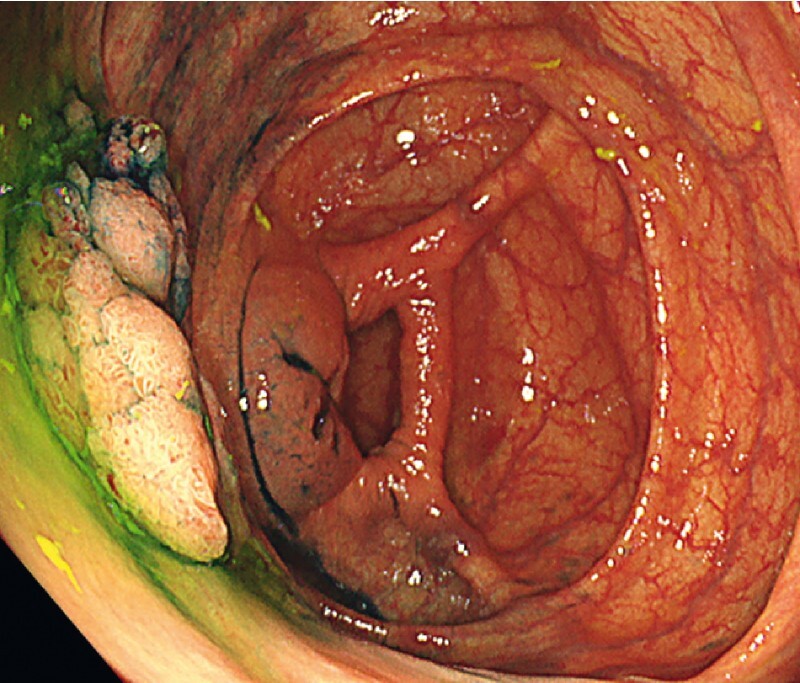
Endoscopic image showing a laterally spreading, granular-nodular, mixed-type tumor, 50 mm in diameter, located in the ascending colon. The lesion was on the gravity side in the supine position and was suitable for gel immersion.

**Fig. 2:**
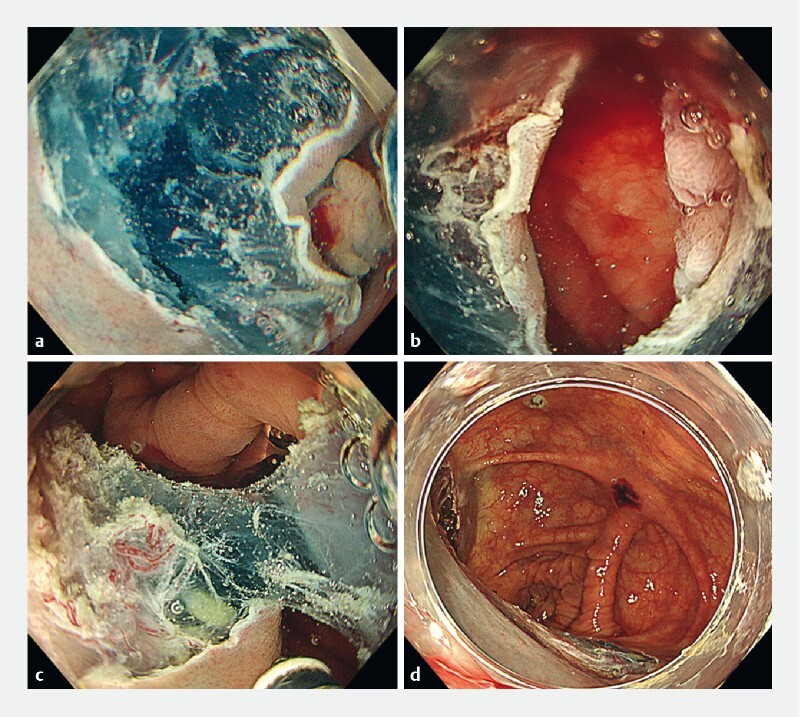
Gel immersion endoscopic submucosal dissection using the tunneling method. **a** A suitable approach to the submucosal layer at the start of tunnel creation owing to the buoyancy of the gel. **b** After tunnel penetration. **c** Smooth mucosal incision and dissection were performed on both sides of the tunnel owing to the buoyancy of the gel. **d** Mucosal defect after gel immersion endoscopic submucosal dissection using the tunneling method.

Endoscopy_UCTN_Code_TTT_1AQ_2AD

## Notes

Endoscopy E-Videos

https://eref.thieme.de/e-videos
*E-Videos* is an open access online section of the journal *Endoscopy* , reporting on interesting cases and new techniques in gastroenterological endoscopy. All papers include a high-quality video and are published with a Creative Commons CC-BY license. Endoscopy E-Videos qualify for HINARI discounts and waivers and eligibility is automatically checked during the submission process. We grant 100% waivers to articles whose corresponding authors are based in Group A countries and 50% waivers to those who are based in Group B countries as classified by Research4Life (see: https://www.research4life.org/access/eligibility/ ). This section has its own submission website at https://mc.manuscriptcentral.com/e-videos
